# Supplementary material for: The genome sequence of a bark-dwelling beetle, Silvanus unidentatus (Olivier, 1790)
Source: Wellcome Open Res. 2025 Mar 18;10:136. [Version 1] doi: 10.12688/wellcomeopenres.23771.1 (PMC11979581; doi:10.12688/wellcomeopenres.23771.1)
Supplement: Peer review history

# Reviewer report: Reviewer response for version 1

**DOI:** 10.21956/wellcomeopenres.26219.r121438

**Published:** 2025-04-08

**Authors:** Henrique Antoniolli

**Affiliations:** 1 Universidade Federal do Rio Grande do Sul (UFRGS), Porto Alegre, Brazil

The present study provides a highly contiguous genome assembly for a bark-dwelling beetle, *Silvanus unidentatus*.

- Background section brings information about its general biology, but lack the rationale for sequencing the genome of this species. The authors should clearly state what motivated the study and how it advances the current knowledge about bark-dwelling beetles. 
- Methods and results are clearly described and presented; including accession numbers for all sequencing data and detailed protocols.
- Authors should also deposit the annotation for mitochondrial genes.

Are sufficient details of methods and materials provided to allow replication by others?

Yes

Is the rationale for creating the dataset(s) clearly described?

No

Are the datasets clearly presented in a useable and accessible format?

Yes

Are the protocols appropriate and is the work technically sound?

Yes

Reviewer Expertise:

Genomics, Evolution

I confirm that I have read this submission and believe that I have an appropriate level of expertise to confirm that it is of an acceptable scientific standard.

# Reviewer report: Reviewer response for version 1

**DOI:** 10.21956/wellcomeopenres.26219.r120909

**Published:** 2025-03-29

**Authors:** Chenyang Cai

**Affiliations:** 1 Chinese Academy of Sciences, Nanjing, China

The paper is very well written, and the genome is highly complete. The identification was also conducted by experts, ensuring high credibility. Here we present a chromosome-level genome sequence for *Silvanus unidentatus*, based on a female specimen from Bookham Common, Leatherhead, England, United Kingdom (Figure 1). The specimen was collected and identified by M.V.L. Barclay under the bark of a dead oak.

**Minor points:**

- *1. S. unidentatus* measures 2.3–2.8 mm in length. should be ' *Silvanus unidentatus* measures 2.3–2.8 mm in length. '

Are sufficient details of methods and materials provided to allow replication by others?

Yes

Is the rationale for creating the dataset(s) clearly described?

Yes

Are the datasets clearly presented in a useable and accessible format?

Yes

Are the protocols appropriate and is the work technically sound?

Yes

Reviewer Expertise:

beetle phylogenomics

I confirm that I have read this submission and believe that I have an appropriate level of expertise to confirm that it is of an acceptable scientific standard.
